# Supplementary material for: Controlling nanowire growth through electric field-induced deformation of the catalyst droplet
Source: Nat Commun. 2016 Jul 29;7:12271. doi: 10.1038/ncomms12271 (PMC4974563; doi:10.1038/ncomms12271)
Supplement: Supplementary Information — Supplementary Figures 1-5, Supplementary Note 1 and Supplementary References. [file ncomms12271-s1.pdf]

**Supplementary Figure 1**

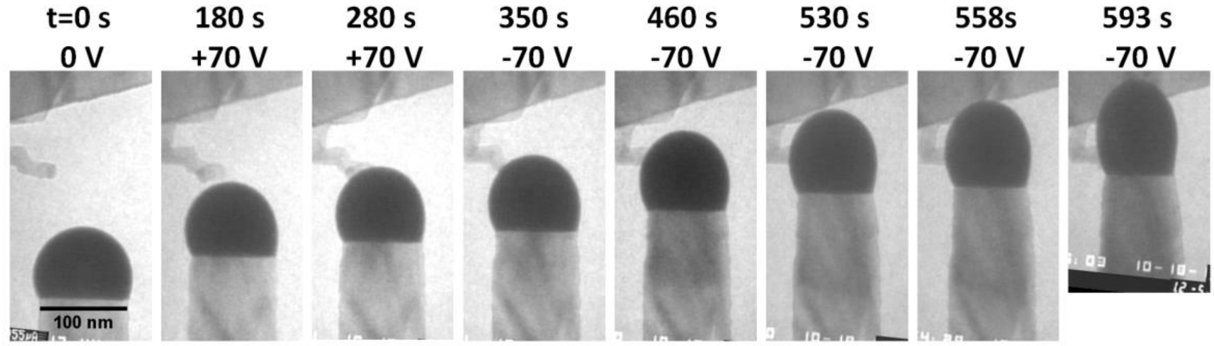


***Growth of nanowires at constant* |V|.** *Sequence of images showing a nanowire growing at 480°C and 1.2×10⁻⁶ Torr of $Si_2H_6$ with times and voltages indicated. As this nanowire grows it approaches the counter electrode and the field (V/distance) and hence deformation both increase. Note the diameter reduction from 100 nm to 75 nm at t=593 s.*

**Supplementary Figure 2**

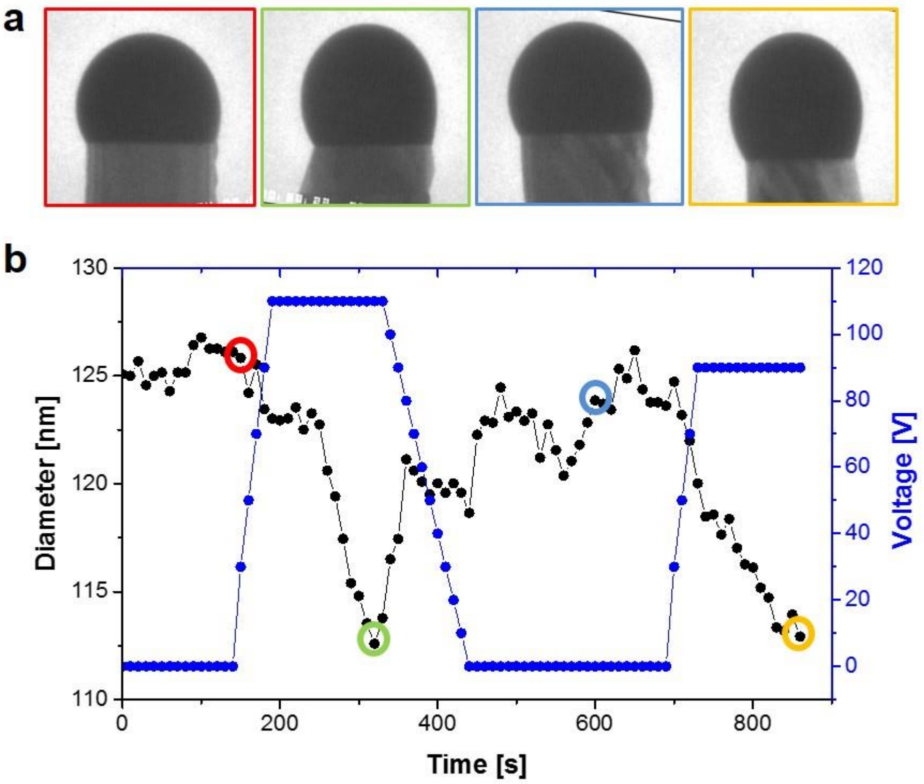


***Growth of nanowires under variable electric field.*** *Nanowire growing at 480ºC and 1.5×10*$^{-5}$ *Torr* $Si_2H_6$ *under an applied voltage between 0 and 110 V.* ***a*** *Image sequence showing droplet deformation and diameter change. When the droplet is stretched, the diameter progressively decreases in order to reestablish the equilibrium contact angle. After removing the electric field, the droplet and the diameter of the wire reacquire the initial shape and size. This shows that the diameter change is reversible and can be used to effectively modulate the nanowire cross section.* ***b*** *Wire diameter (black) and applied voltage (blue) vs. time. The small fluctuations in diameter uncorrelated with V are caused by the sidewall sawtooth geometry [[1]]. Colored circles identify the data corresponding to images in* ***a****.*

**Supplementary Figure 3**

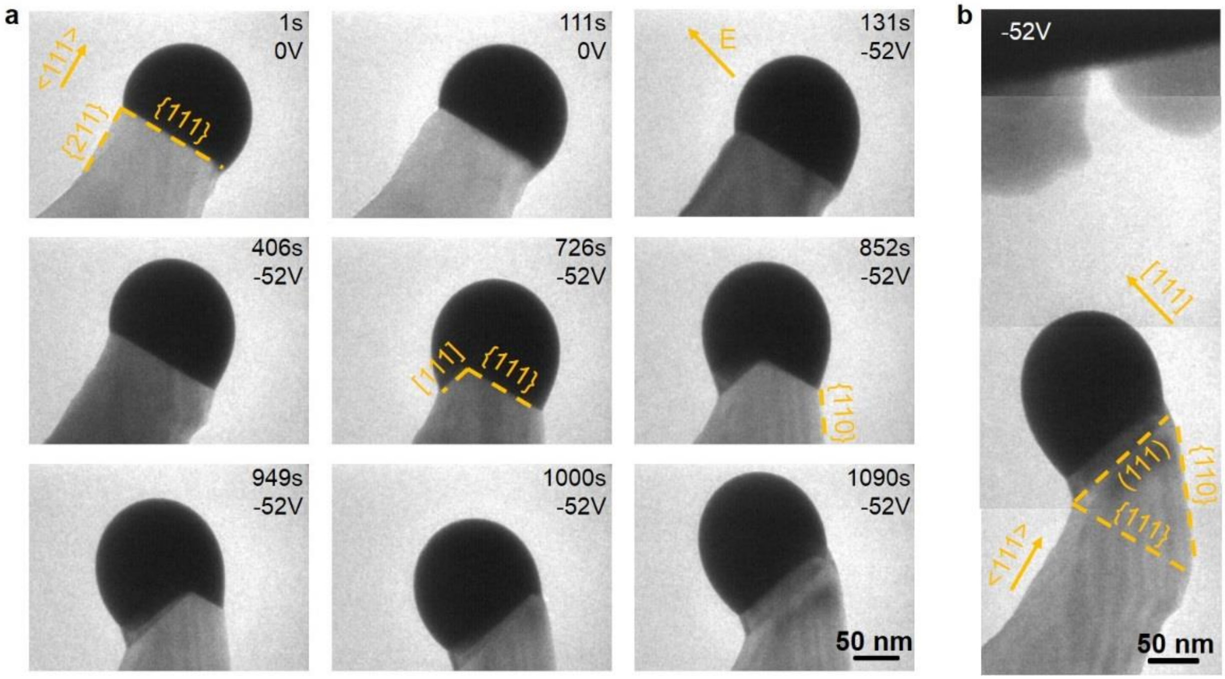


***Nanowire kinking directed by electric field. a*** *Image sequence showing a nanowire growing in a <111> direction at 510ºC and ~1.6 × 10*$^{-5}$ *Torr of* $Si_2H_6$ *. When a field is applied in the [111] direction (at ~70*$^{o}$ *to the growth direction), the nanowire gradually kinks to this direction.* ***b*** *Montage showing the entire wire from* ***a****.*

**Supplementary Figure 4**

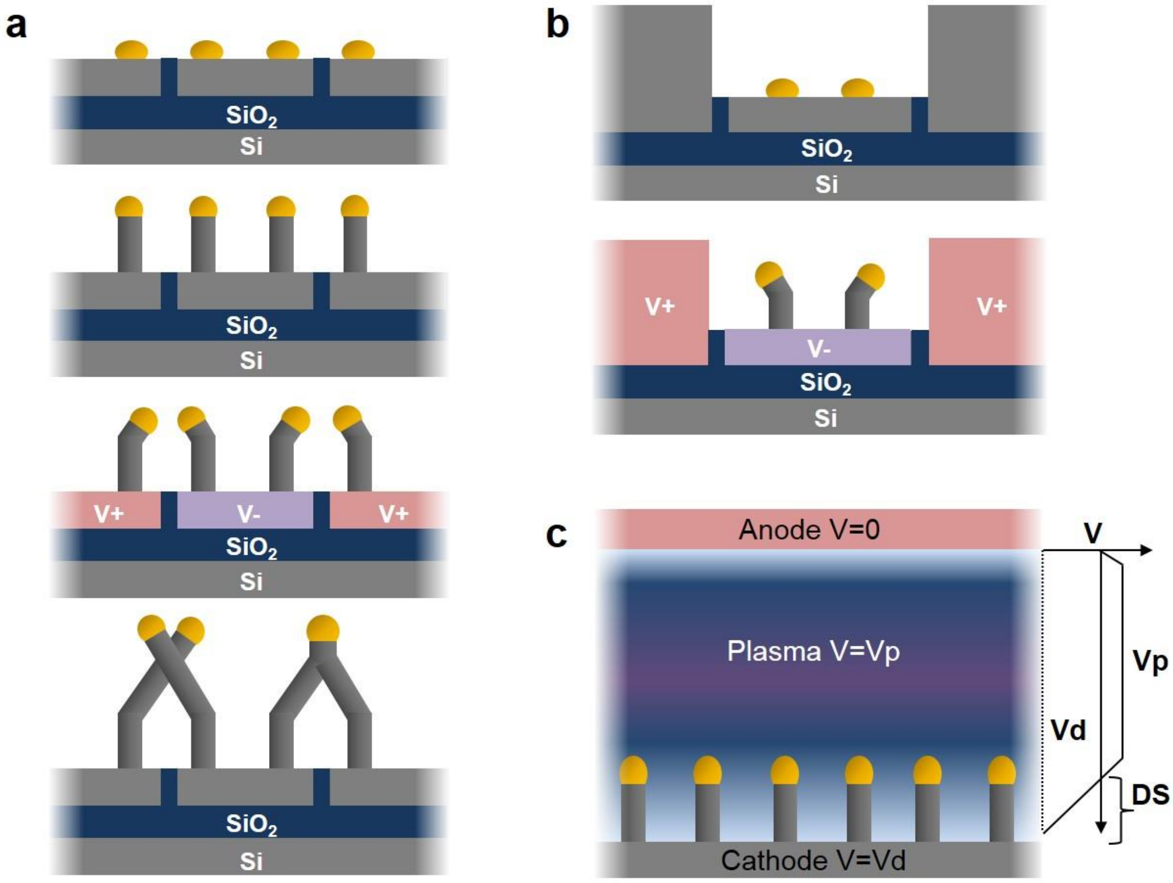


**Nanowire growth opportunities.** ***a*** *Sequence of schematics showing the formation of complex nanowire structures by applying a voltage between nanowires grown on patterned electrodes. The E-field induces nanowires to kink and form X (for slightly misaligned wires) or Y (for aligned wires) nanostructures.* ***b*** *Sequence of schematics showing nanowire growth on polarized NEMS structures.* ***c*** *Schematic showing the growth of nanowires in a PECVD reactor. The electric field generated at the Debye sheath (DS) can be used to deform the catalyst droplet.*

**Supplementary Figure 5**

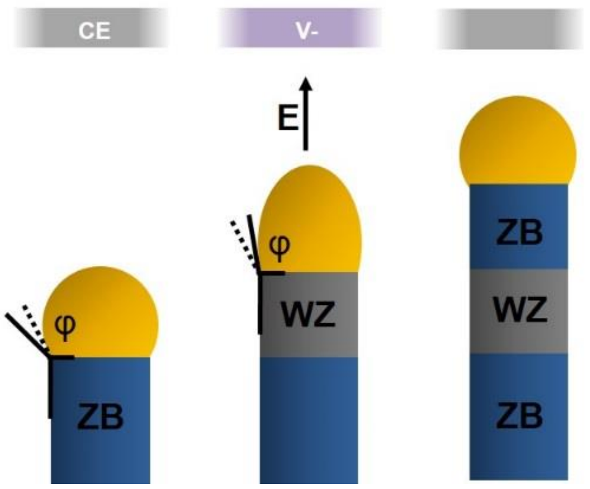


***Field induced phase switching in GaAs.*** *Schematics illustrating the concept of phase switching* (wurtzite versus zinc blende structure) *in GaAs by changing the droplet/nanowire contact angle with an electric field. Dashed line represents the critical angle at which phase switching occurs.*

## Supplementary Note 1

**Implementation in a conventional reactor and possible applications.** In Supplementary Figure 4, we illustrate three methods that could allow implementation in a conventional reactor to create complex nanostructures.

a) Field-directed nanowire growth using electrodes built into the growth substrate. In Supplementary Figure 4a we show an example of how NWs can be grown on a patterned substrate that allows the polarization of a row or group of nanowires. The electrostatic attraction between wires is used to form X or Y shaped nanostructures depending on the initial wire locations. Self-assembled nanowire networks has been sought as platforms for future advanced electronics [[2]] including Majorana-fermion-based devices [[3]].

b) In a similar way nanowires can be grown on nano-electromechanical (NEMS) structures integrated on Si wafers [[4,5,6]]. The nanostructure can be polarized to direct the growth (Supplementary Figure 4b)

c) It is well documented that growth of carbon nanotubes (CNTs) in conventional PECVD reactors leads to CNT alignment due to the electric field generated in the Debye sheath (DS) [[7,8,9,10]]. The same concept can be applied to generate an electric field during nanowire growth. The advantage of using a plasma is that the E-field around the nanowires is not determined by the distance and potential difference between nanowires and CE, but by the thickness of the DS and the difference between the plasma potential (Vp) and the wire potential (Vd) (see Supplementary Figure 4c). Typical DS thicknesses are in the order of a few μm to a few mm. The DS thickness as well as Vp can be tuned by changing Vd and the reactor geometry. This method would allow us to generate an electric field of several V/μm oriented parallel to the wire growth. Forming a DC plasma requires at least 500 V, but it can be sustained at lower voltage by means of a RF source or by a source of electrons.

## Supplementary References

[1] Ross, F.M., Tersoff, J., Reuter, M.C., Sawtooth faceting in silicon nanowires. *Physical Review Letters*, **95**(14), 146104 (2005).

[2] Dai, X., Dayeh, S.A., Veeramuthu, V., Larrue, A., Wang, J., Su, H. and Soci, C., Tailoring the vapor–liquid–solid growth toward the self-assembly of GaAs nanowire junctions. *Nano letters*, **11**(11), 4947-4952 (2011).

[3] Car, D., Wang, J., Verheijen, M.A., Bakkers, E.P. and Plissard, S.R., Rationally Designed Single-Crystalline Nanowire Networks. *Advanced Materials*, **26**(28), 4875-4879 (2014).

[4] Islam, M.S., Sharma, S., Kamins, T.I. and Williams, R.S., Ultrahigh-density silicon nanobridges formed between two vertical silicon surfaces. *Nanotechnology*, **15**(5), L5 (2004).

[5] Sharma, S., Kamins, T.I., Islam, M.S., Williams, R.S. and Marshall, A.F., Structural characteristics and connection mechanism of gold-catalyzed bridging silicon nanowires. *Journal of crystal growth*, **280**(3), 562-568 (2005).

[6] Chaudhry, A., Ramamurthi, V., Fong, E. and Islam, M.S., Ultra-low contact resistance of epitaxially interfaced bridged silicon nanowires. *Nano letters*, **7**(6), 1536-1541 (2007).

[7] Ren, Z.F., Huang, Z.P., Xu, J.W., Wang, J.H., Bush, P., Siegal, M.P. and Provencio, P.N., Synthesis of large arrays of well-aligned carbon nanotubes on glass. *Science*, **282**(5391), 1105-1107 (1998).

[8] Chhowalla, M., Teo, K.B.K., Ducati, C., Rupesinghe, N.L., Amaratunga, G.A.J., Ferrari, A.C., Roy, D., Robertson, J. and Milne, W.I., Growth process conditions of vertically aligned carbon nanotubes using plasma enhanced chemical vapor deposition. *Journal of Applied Physics*, **90**(10), 5308-5317 (2001).

[9] Hofmann, S., Csanyi, G., Ferrari, A.C., Payne, M.C. and Robertson, J., Surface diffusion: the low activation energy path for nanotube growth. *Physical Review Letters*, **95**(3), 036101 (2005).

[10] Hofmann, S., Ducati, C., Robertson, J. and Kleinsorge, B., Low-temperature growth of carbon nanotubes by plasma-enhanced chemical vapor deposition. *Applied Physics Letters*, **83**(1), 135-137 (2003).
